# Supplementary material for: LC-MS Based Phytochemical Profiling towards the Identification of Antioxidant Markers in Some Endemic Aloe Species from Mascarene Islands
Source: Antioxidants (Basel). 2022 Dec 26;12(1):50. doi: 10.3390/antiox12010050 (PMC9854647; doi:10.3390/antiox12010050)
Supplement: Supplementary file 1 [file antioxidants-12-00050-s001.zip › antioxidants-2065220-supplementary.pdf]

LC-MS based phytochemical profiling towards the identification of antioxidant markers in some endemic *Aloe* species from Mascarene Islands

Célia Breaud [1, †], Laura Lallemand [2, †], Gary Mares [2], Fathi Mabrouki [1], Myriam Bertolotti [1], Charlotte Simmler [1], Stéphane Greff [1], Morgane Mauduit [1], Gaëtan Herbette [3], Eldar Garayev [4], Christophe Lavergne [5], Maya Cesari [2], Sok-Siya Bun-Llopet [1], Béatrice Baghdikian [1] and Elnur Garayev [1]*

[1] Aix Marseille Univ, CNRS 7263, IRD 237, Avignon Université, IMBE, 27 blvd Jean Moulin, Service of Pharmacognosy, Faculty of Pharmacy, 13385 Marseille, France.
[2] CYROI, Plateforme de recherche, Cyclotron Réunion Océan Indien, La Réunion, France
[3] CNRS, Centrale Marseille, FSCM, Spectropole, Aix Marseille Université, Campus de St Jérôme-Service 511, 13397 Marseille, France.
[4] Department of General and Toxicological Chemistry, Azerbaijan Medical University, AZ1001, Baku, Azerbaijan
[5] CBNM Conservatoire Botanique National de Mascarin, 2, rue du Père Georges, Les Colimaçons, 97436 Saint-Leu, La Réunion, France
* Correspondence: elnur.garayev@imbe.fr
† These authors contributed equally to this work.

Table S1. MZmine parameters

Figure S1. DPPH inhibitory activity of inactive extracts.

Table S1. MZmine parameters

Module	*Parameters*
Raw data methods > Raw data import	Importation of all .mzXML files
Raw data methods > Mass detection	Scans MS level : **1**<br>Mass detector : **centroid**<br>Noise level : **2.0E2**; **1.5E2**
Raw data methods > Mass detection	Scans MS level : **2**<br>Mass detector : **centroid**<br>Noise level : **1.3E1**
Feature detection > LC-MS > ADAP chromatogram builder	Scans MS level : **1**<br>Min group group size in # of scans : **4**<br>Group intensity threshold : **1.0E3**<br>Min highest intensity : **1.0E3**<br>Scan to scan accuracy (m/z) : **10 ppm**
Feature detection > Chromatogram resolving > Local minimum resolver	Chromatographic threshold : **20%**<br>Minimum search range RT/Mobility (absolute) : **0.10**<br>Minimum relative height : **15%**<br>Minimum absolute height : **2.0E3**; **1.0E3**<br>Min ratio of peak top/edge : **3**<br>Peak duration range (min/mobility) : **0.01 -1.00**<br>Min # of data points : **3**
Feature list methods > Isotopes > 13C isotope filter	m/z tolerance : **10 ppm**<br>Retention time tolerance : **0.1 absolute (min)**<br>Mobility tolerance : **unchecked**<br>Monotonic shape : **unchecked**<br>Maximum charge : **2**<br>Representative isotope : **most intense**<br>Never remove feature with MS2 : **checked**
Feature list methods > Alignment > Join aligner (within replicates)	m/z tolerance : **10 ppm**<br>Weight for m/z : **75**<br>Retention time tolerance : **0.2 absolute (min)**<br>Weight for RT : **25**<br>Mobility tolerance : **unchecked**<br>Mobility weight : **1.00**<br>Require same charge state : **checked**<br>Require same ID : **unchecked**<br>Compare isotope pattern : **unchecked**<br>Compare spectra similarity : **unchecked**
Feature list methods > Processing > Assign MS^2 to features	Retention time tolerance : **0.1 absolute (min)**<br>MS1 to MS2 precursor tolerance (m/z) : **10 ppm**<br>Limit by RT edges : **unchecked**<br>Combine MS/MS spectra (TIMS) : **unchecked**<br>Lock to feature mobility range : **unchecked**<br>Minimum merged intensity : **unchecked**
Feature list methods > Feature list filtering > Feature list rows filter (within replicates)	Minimum features in a row (abs or %) : **3**<br>Retention time : **0.6 - 30.0 min (auto range)**<br>Features with MS2 scans : **checked**

Feature list methods > Alignment > Join aligner (across all samples)	m/z tolerance : **10 ppm**<br>Weight for m/z : **75**<br>Retention time tolerance : **0.2 absolute (min)**<br>Weight for RT : **25**<br>Mobility tolerance : **unchecked**<br>Mobility weight : **1.0**<br>Require same charge state : **checked**<br>Require same ID : **unchecked**<br>Compare isotope pattern : **unchecked**<br>Compare spectra similarity : **unchecked**
Feature list method > Feature list filtering > Feature list rows filter (across all samples)	Minimum features in a row (abs or %) : **3**<br>Retention time : **0.6 - 30.0 min (auto range)**<br>Features with MS2 scans : **checked**<br>Reset the feature number ID : **checked**
Feature list method > Export feature list > GNPS - feature based molecular networking	Merge MS/MS (experimental) : **checked**<br>Select spectra to merge : **across samples**<br>m/z merge mode : **most intense**<br>intensity merge mode : **maximum intensity**<br>Expected mass deviation : **5 ppm**<br>Cosine threshold : **70 %**<br>Signal count threshold : **20 %**<br>Isolation window offset : **0.0**<br>Isolation window width (m/z): **3.0**<br>Filter rows : **ALL**<br>Feature intensity : **Peak area**<br>CSV export : **ALL**<br>Submit to GNPS : **unchecked**<br>Open folder : **unchecked**

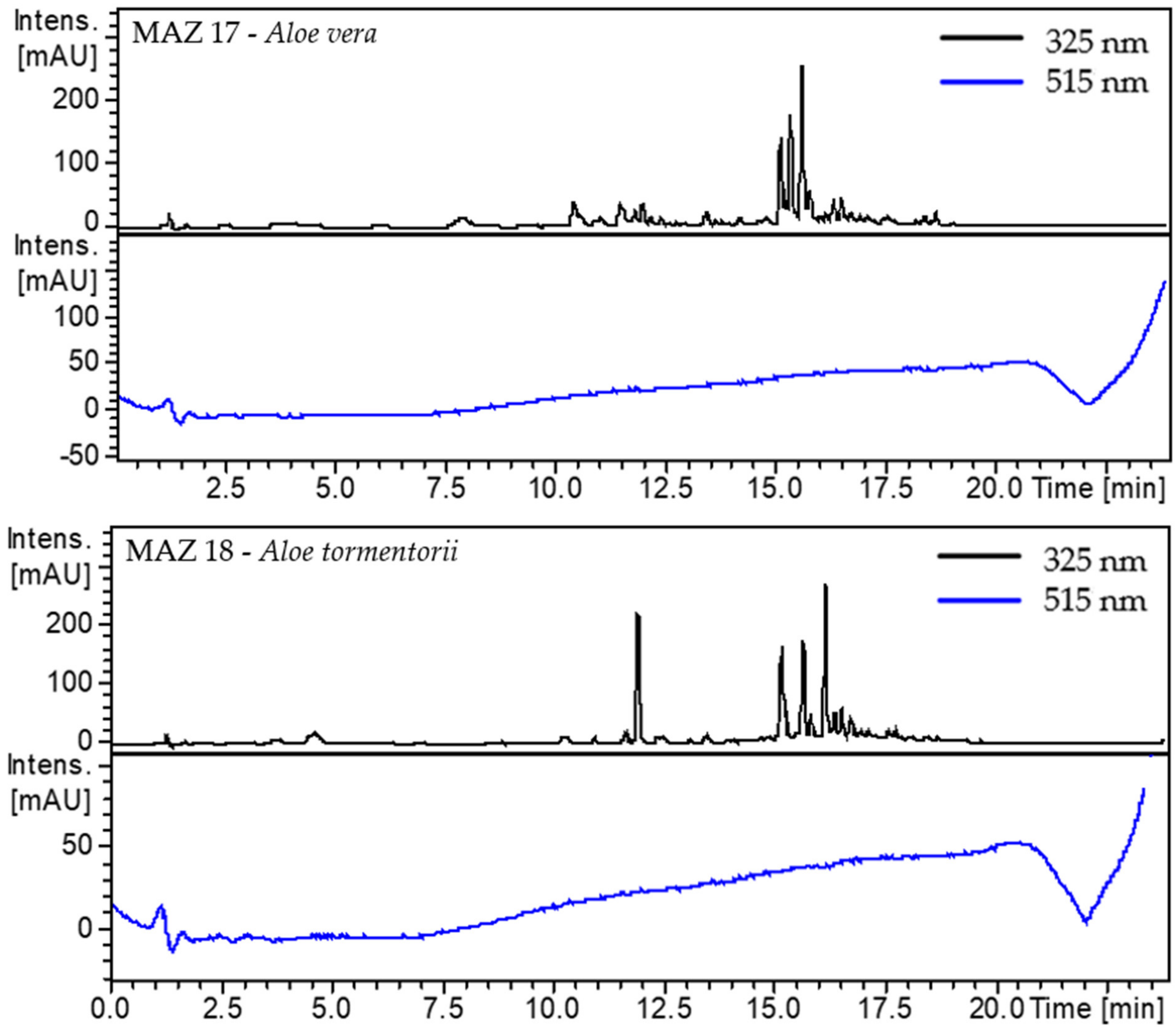

Figure S1. DPPH inhibitory activity of inactive extracts.
